# Experimental Study of Wear Mechanisms of Cemented Carbide in the Turning of Ti6Al4V

**DOI:** 10.3390/ma12172822

**Published:** 2019-09-02

**Authors:** Sara Saketi, Stina Odelros, Jonas Östby, Mikael Olsson

**Affiliations:** 1 Materials Science, Dalarna University, SE-791 88 Falun, Sweden; 2 Ångström Tribomaterials Group, Uppsala University, SE-581 83 Uppsala, Sweden; 3 R&D, Sandvik Coromant, SE-12680 Stockholm, Sweden

**Keywords:** turning, cemented carbide, Ti6Al4V, attrition wear, diffusion wear, SEM, EDS

## Abstract

Titanium and titanium alloys such as Ti-6Al-4V are generally considered as difficult-to-machine materials. This is mainly due to their high chemical reactivity, poor thermal conductivity, and high strength, which is maintained at elevated temperatures. As a result, the cutting tool is exposed to rather extreme contact conditions resulting in plastic deformation and wear. In the present work, the mechanisms behind the crater and flank wear of uncoated cemented carbide inserts in the turning of Ti6Al4V are characterized using high-resolution scanning electron microscopy (SEM), energy-dispersive X-ray spectroscopy (EDS), and high-resolution Auger electron spectroscopy (AES).The results show that, for combinations of low cutting speeds and feeds, crater and flank wear were found to be controlled by an attrition wear mechanism, while for combinations of medium to high cutting speeds and feeds, a diffusion wear mechanism was found to control the wear. For the latter combinations, high-resolution SEM and AES analysis reveal the formation of an approximately 100 nm thick carbon-depleted tungsten carbide (WC)-layer at the cemented carbide/Ti6Al4V interface due to the diffusion of carbon into the adhered build-up layers of work material on the rake and flank surfaces.

## 1. Introduction

In machining, tribochemical and mechanical properties of the work and tool materials and the cutting conditions (cutting speed, cutting feed, etc.) affect the tool wear, which, in turn, affect the efficiency of the machining operation and the surface roughness of the machined surface. As a result, in order to improve the robustness of a machining operation and minimize the machining costs, it is very necessary to improve the wear resistance of the cutting tool and reliably predict the tool-life. In machining, tool wear mainly takes place along the cutting edge and on the flank and rake faces [1,2]. In addition to deformation, wear along the cutting edge is, in general, caused by cracking and micrchipping. Wear on the flank face is caused by the sliding contact between the tool flank and the newly machined workpiece surface while wear on the rake face, frequently resulting in a crater, is caused by the sliding contact between the rake face and the formed chip. In general, flank wear is controlled by a mechanical wear mechanism, e.g., abrasion, while crater wear, due to the higher temperatures on the rake face, is controlled by a tribochemical wear mechanism, e.g., diffusion wear or solution wear. The former mechanism has typically been considered to have a wear rate proportional to sliding velocity after a fundamental model by Archard [3]. The latter mechanism can be considered rate-limited by temperature according to a model by Usui et al. [4]. A corresponding wear mechanism with an Archard-type, exponential, temperature dependence has been specifically considered for Ti6Al4V by Zanger and Schulze [5].

Recent articles by Kaplan et al. [6] and Odelros et al. [7] establish decarburization of tungsten carbide (WC) in contact with Ti-alloy both through machining experiments and diffusion couples. The work by Kaplan et al. [6] finds plastic deformation of the binder to be significant at higher cutting velocities. The work by Odelros et al. [7] provides a measured wear rate at cutting speed 70 m/min and further notes a possibility that the binder phase could melt at higher cutting speeds, which would contribute significantly to the wear rate. In a work by Hatt et al. [8], a diffusion couple interface between cemented carbide and Ti-alloy is investigated in detail, confirming formation of carbides from the alloy.

To link a wear model such as that by Zanger and Schulze [5] to a physical wear mechanism, an activation energy for a known rate-limiting reaction could be used. Alternatively, wear rates depending on temperatures could be used to estimate an activation energy, which could be compared to possible reactions. A recent calculation of such possible reactions underlying decarburization of WC in contact with Ti6Al4V has been made by Edin et al. [9], highlighting the interest for more experimental data on the formation of carbon-depleted layers in the material interfaces.

**Figure 1:**
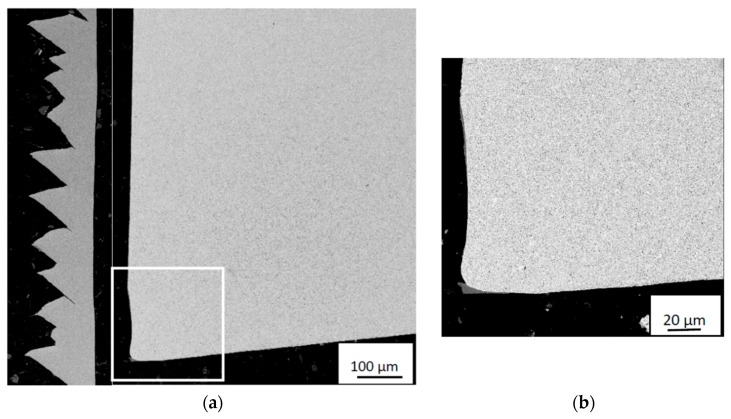
Cross-sections of Ti6Al4V chip and cemented carbide insert (to scale) illustrating the chip thickness and morphology with respect to the size of the flank and the crater on the insert rake face. (**a**) At lower and (**b**) at higher magnification. The pronounced segmented nature of the chip generates cyclic stresses which promote surface fatigue of the cutting edge.

Ti-6Al-4V is an α + β alloy which is the most employed titanium alloy in aerospace applications due to the corrosion resistance, the high strength-to-weight ratio and the fatigue properties at elevated temperature [10,11]. On the other hand, Ti6Al4V shows machining challenges which is mainly due to a combination of properties including:

- the high temperature strength of titanium and pronounced work hardening ability have a negative impact on the chip formation mechanisms [12];
- the low heat conductivity of titanium promotes high temperatures at the cutting edge and the rake face [13,14,15];
- the segmented nature of the chips generates cyclic stresses which promote surface fatigue of the cutting edge [16,17], cf. Figure 1;
- the small chip/tool contact length and the very thin flow zone between the chip and rake face result in high stresses and high temperatures in close connection to the cutting edge [18], cf. Figure 1;
- the high chemical reactivity between titanium and the tool material promotes strong bonding and diffusion wear at the rake face resulting in a crater [19,20,21,22], cf. Figure 1.

An illustration of the chip and cutting zone is given in Figure 1 for an orthogonal cutting process after 0.5 min cutting time with cutting parameters *v_c_* 115 m/min, *f_n_* 0.2 mm/rev, *a_p_* 3 mm.

Generally, titanium and titanium alloys are machined with uncoated WC-Co cemented carbide inserts [23]. It is well accepted that the dominant gradual wear mechanism of cemented carbide during turning of titanium and titanium alloys is controlled by diffusion at low and medium cutting speeds (30–90 m/min) [24,25,26,27,28] while at higher cutting speeds severe plastic deformation leading to edge depression and micro or macro fracture of the cutting edge region occurs [29,30,31,32], and some authors [23,33] believe that 60 m/min is the cutting speed which higher than that limit the lifetime of cemented carbide cutting tool due to the severe plastic deformation. The aim of the present study was to examine the wear characteristics of cemented carbide in the turning of a Ti6Al4V alloy using high-resolution scanning electron microscopy, energy-dispersive X-ray spectroscopy, and high-resolution Auger electron spectroscopy (AES). The contact conditions occurring at the tool crater/chip and tool flank/work material interfaces are discussed and interpreted as the wear characteristics of the crater and flank wear respectively. Measured wear rates for multiple machining operations are reported.

## 2. Experimental

### 2.1. Materials

**Figure 2:**
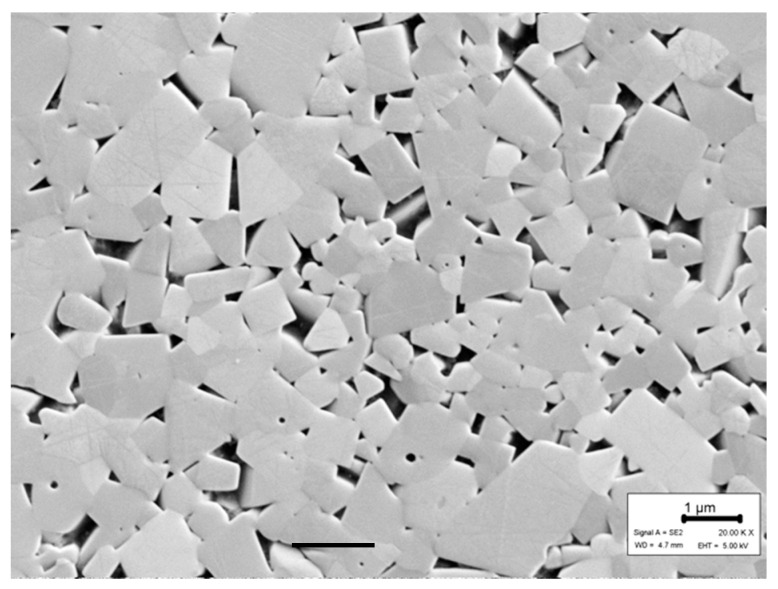
Microstructure of the cemented carbide grade investigated. WC phase appears bright, Co phase appears dark.

The work material used in the present study was a mill-annealed bar of Ti6Al4V alloy, grade 5, supplied by Timet, see Table 1. The cemented carbide grade used was H13A (Sandvik Coromant, Sandviken, Sweden), a commercially available uncoated WC–Co grade which combines good wear resistance and toughness for the turning of heat resistant steels and titanium alloys at moderate cutting speeds and feeds. Figure 2 shows the microstructure of the cemented carbide grade while Table 2 shows the chemical composition and some mechanical properties.

### 2.2. Turning Tests

**Figure 3:**
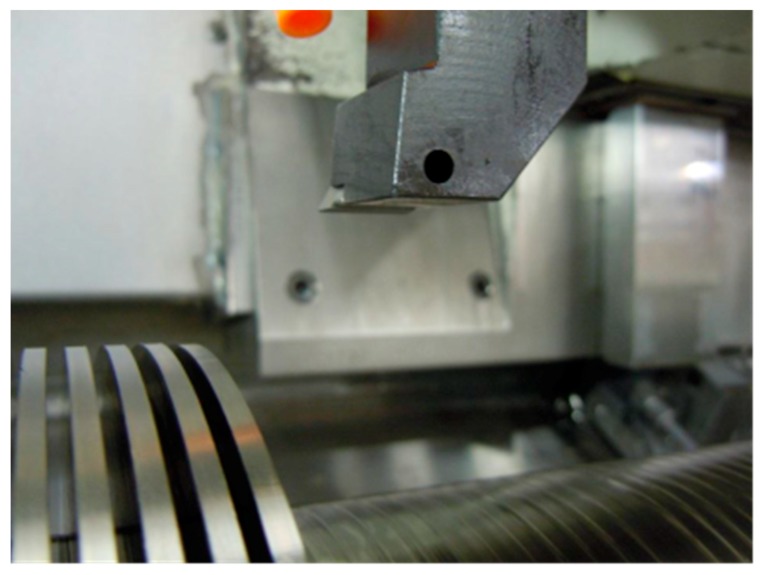
Test set-up for orthogonal turning tests using a flanged Ti6Al4V work piece.

A commercial George Fischer CNC lathe (Schaffhausen, Switzerland), NDM-17/125 were used for semi-orthogonal turning tests using uncoated flat-faced cemented carbide inserts (ISO code: TCMW 16T304). The cutting tool holder used was a STFCR2525M 16, giving no rake angle and a near-straight entrance angle. To get a close to orthogonal cutting approach it was decided to perform a radial turning operation using a pre-prepared workpiece with flanges 3 mm wide and 3 mm apart, and with a 30 mm depth along the cylindrical surface, see Figure 3. The initial workpiece diameter was 180 mm. Before constructing the flanged structure, a 1 mm thick top layer of the workpiece material was removed to get a clean and smooth surface. Cutting parameters for the main tests series were; cutting speed *v_c_*: 90, 100 and 115 m/min, feed rate *f_n_*: 0.05, 0.10, 0.15 and 0.2 mm/rev, cutting depth *a_p_*: 3.0 mm. Complementary tests were performed also at lower cutting speeds, i.e., 45 and 60 m/min. In order to follow the evolution of flank and crater wear over time, turning experiments were run for 0.5, 1, 2 and 3 min (when possible). All tests were repeated twice, giving a total of 6–8 measurements for each wear rate estimation. All tests were performed with a 6% solution of a semi-synthetic coolant, Hocut B50S (Houghton International, Norristown, PA, USA), with a pH of 9.5 and the typical flow rate of 200 L/min. 

### 2.3. Post Test Evaluation

#### 2.3.1. Tool Wear

After the turning tests, the rake face wear of the cutting inserts was evaluated using an Alicona Infinite Focus 3D optical surface profilometer (Graz, Austria). By comparing the 3D surfaces of a worn cutting edge with an unworn reference cutting edge, respectively, the wear (volume loss) of the worn rake face was evaluated. In order to exclude the influence from adhered work material, the inserts were etched in 40% hydrofluoric acid for 20 min. at room temperature before performing the measurements. It could be noted that the plastic deformation of the cutting edge was not accounted for when measuring the crater volumes. The flank wear, i.e., the width of the flank, of the cutting inserts were measured using scanning electron microscopy.

To prepare cross-section specimens, the inserts were cut perpendicular to the cutting edge in the centre of the crater wear region, using a diamond cutting disc. The cross-section samples were then hot mounted in a conductive epoxy molding compound and then ground on Piano disc to get a flat surface. Finally, the surface was polished with 6, 3 and 1 μm diamond spray on polishing discs.

#### 2.3.2. Wear Mechanisms

The surface degradation and wear characteristics of the cutting inserts were characterized using high-resolution scanning electron microscopy (FEG-SEM, Zeiss, Oberkochen, Germany, Ultra 55). The element composition of the worn surfaces was analyzed using energy-dispersive X-ray spectroscopy (EDS, Oxford Inca Energy, Abingdon, UK). in order to limit the interaction volume and increasing the sensitivity to light elements the SEM and EDS analyses were preferably performed using an acceleration voltage of 3–5 kV resulting a low typically 3–5 keV primary electron energy.

An Ulvac-Phi 700 Xi Scanning Auger Nano prope (Ulvac-PHI, Chigasaki, Japan) was used to investigate the diffusion of specific elements of tool material into the work material. The AES analysis was performed using an accelerating voltage of 10 kV and a primary beam current of 10 nA. Depth profiling were performed using 2.0 kV Ar^+^ ion sputtering. The sputter rate was 17.1 nm/min measured on a Ta_2_O_5_ reference sample with known thickness (100 nm). In order to evaluate the Auger depth profile data, computer software from PHI-Matlab (version 9.3) was used. To evaluate the mechanical and adhesion strength of the diffusion layer formed in the crater area, scratch testing was performed at normal loads 0.01 N and 0.05 N using a sharp diamond stylus (radius 7.5 μm) and a commercial scratch test equipment, (CSM Revetest, Peseux, Switzerland).

## 3. Results

### 3.1. Contact Conditions at the Cutting Insert/Work Material Interface

**Figure 4:**
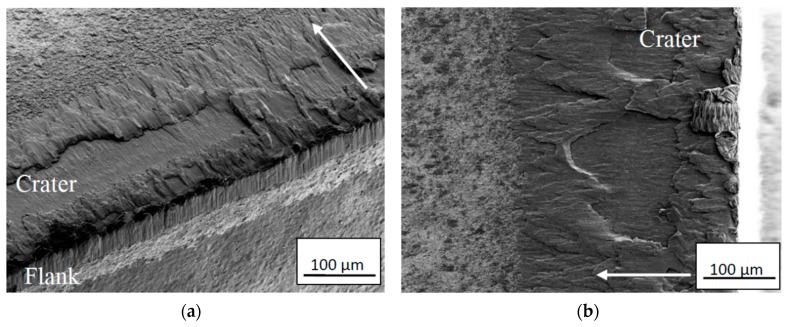
Wear characteristics of cemented carbide insert showing the crater and flank wear region (**a**) and crater (**b**). Cutting parameters: *v_c_*: 90 m/min, *f_n_*: 0.2 mm, *t*: 0.5 min. The arrow shows the chip flow direction. Note the transfer of work material to the cutting edge region.

**Figure 5:**
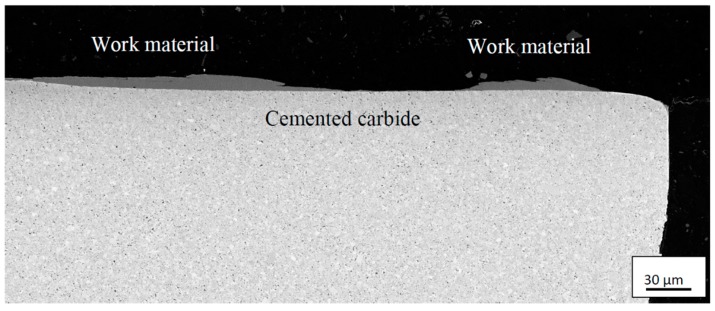
Cross-section of cemented carbide insert showing the crater, partly filled with adhered work material, and flank wear regions. Cutting parameters: *v_c_*: 90 m/min, *f_n_*:0.2 mm/rev, *t*: 0.5 min.

Figure 4 shows a worn cemented carbide cutting insert after 0.5 min turning in the Ti6Al4V alloy using a cutting speed of *v_c_*: 90 m/min, and feed rate of *f_n_*: 0.2 mm/rev. As can be seen, the cutting insert shows both crater wear and flank wear as well as adhered work material on the rake face and the flank wear land. Figure 5 shows a polished cross-section of the corresponding cutting edge, revealing a significant amount of adhered work material within the crater. Thus, in order to measure the crater wear volume the adhered work material has to be removed.

### 3.2. Wear Characteristics

**Figure 6:**
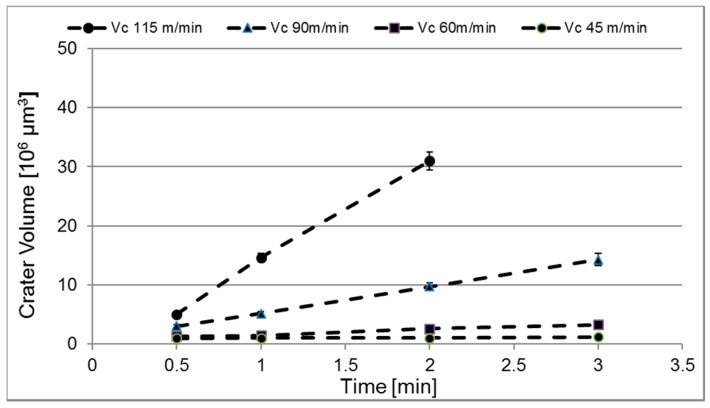
Crater wear vs time at a feed rate of *f_n_* = 0.15 mm/rev and a cutting speed of *v_c_* = 45, 60, 90 and 115 m/min. Above 2 min the combination *f_n_* = 0.15 mm/rev and *v_c_* = 115 m/min results in extensive plastic deformation and macroscopic cracking.

**Figure 7:**
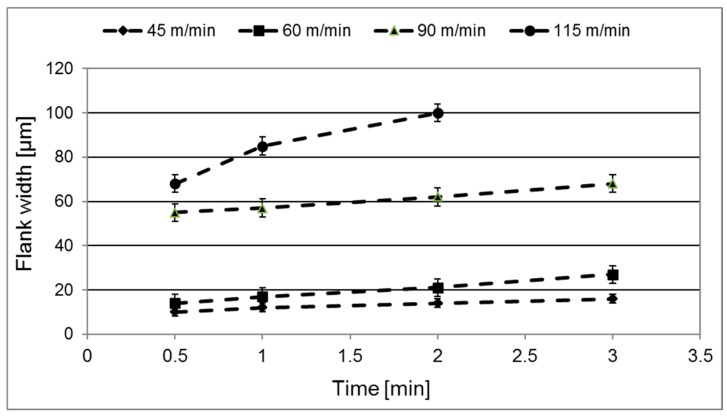
Flank wear vs time at a feed rate of *f_n_* = 0.15 mm/rev and a cutting speed of *v_c_* = 45, 60, 90 and 115 m/min. Above 2 min the combination *f_n_* = 0.15 mm/rev and *v_c_* = 115 m/min results in extensive plastic deformation and macroscopic cracking.

**Figure 8:**
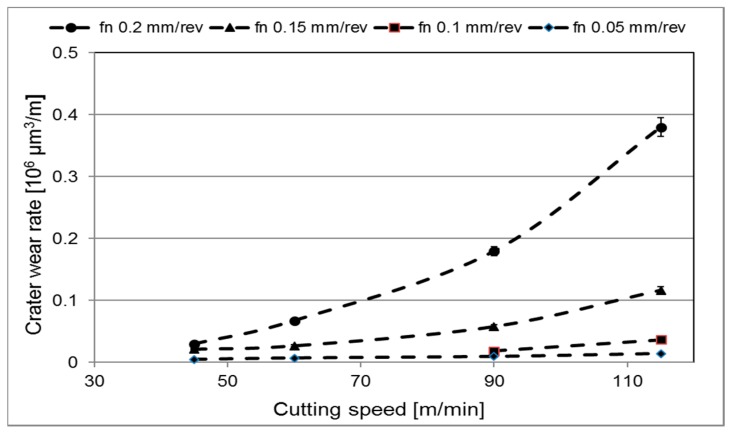
Calculated crater wear rate vs cutting speed *v_c_* for the different feed rates evaluated, *f_n_* = 0.05, 0.10, 0.15, and 0.20 mm/rev.

Figure 6 shows the evolution of crater wear with time during turning for the cutting speeds evaluated using a feed of 0.15 mm/revolution. As can be seen, for all cutting speeds the crater wear volume increases linearly with increasing cutting time. Also, the figure clearly illustrates the strong influence of cutting speed on the crater wear rate, e.g., an increase in cutting speed from 60 to 115 m/min results in a 25 times higher crater wear rate. Similar trends were observed for the other feed rates evaluated. Figure 7 shows the corresponding evolution of flank wear (maximum flank width) with time. Figure 8 shows the normalized crater wear rate in µm^3^/m for the different combinations of cutting feed and cutting speed evaluated. Note that the wear rate expressed in this unit is dependent on the geometry of the specific machining operation. As expected, the crater wear rate increases significantly with increasing feed and speed. This is in good agreement with earlier findings, see e.g., the review paper by Ezugwu E.O. and Wang Z.M. [20]. The observed increases in crater wear rate with increasing cutting speed or cutting feed could agree with a wear mechanism rate-limited by temperature in such a way that has been suggested by e.g., Zanger et al. [5] for titanium machining. Regardless of the underlying wear mechanism, combinations of high cutting feeds and high cutting speeds result in very short tool lives.

**Figure 9:**
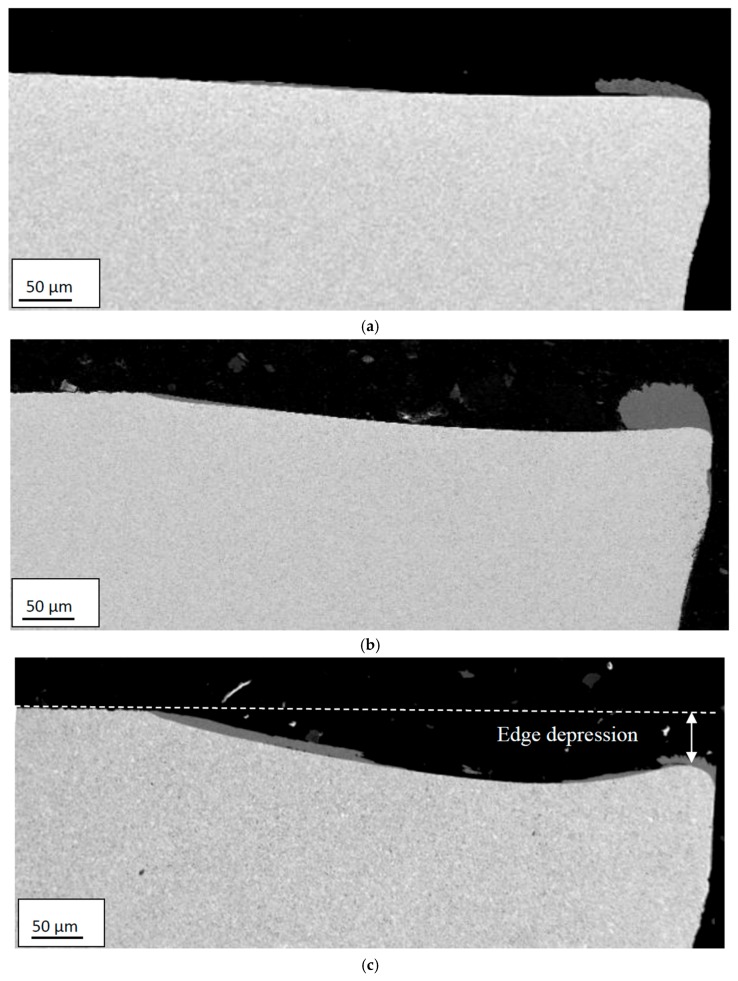
Cross-sections illustrating the influence of time on the resulting crater and flank wear characteristics. (**a**) 1 min; (**b**) 2 min; (**c**) 3 min. Cutting speed: 90 m/min, Feed rate: 0.2 mm/rev. Note the pronounced edge depression observed at the high cutting speed.

**Figure 10:**
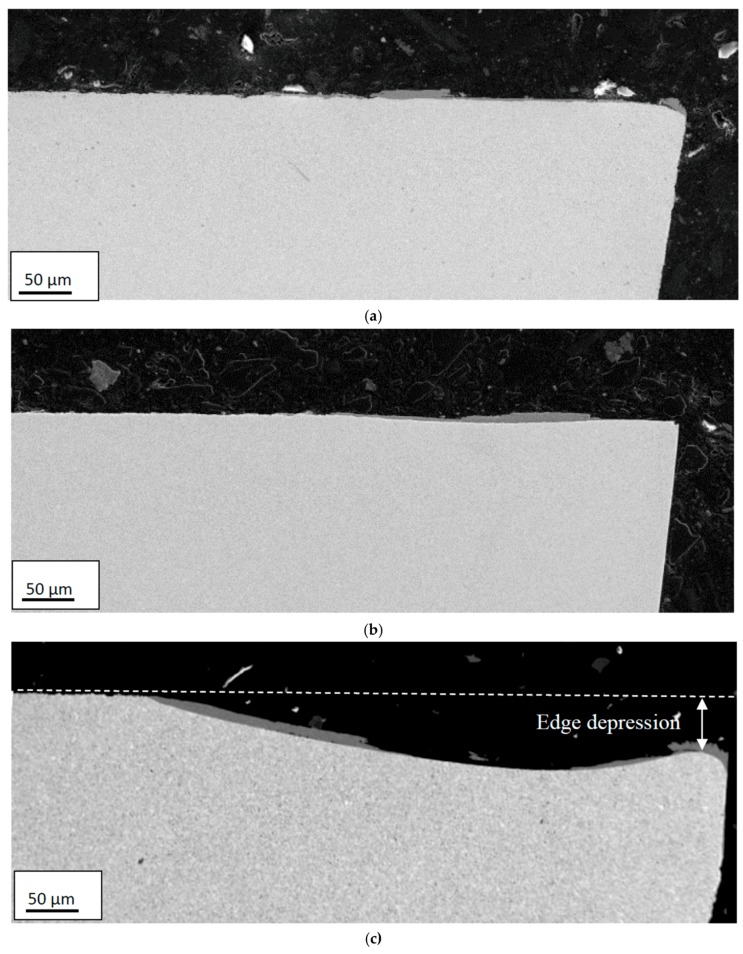
Cross-sections illustrating the influence of cutting speed on the resulting crater and flank wear characteristics. (**a**) 45 m/min; (**b**) 60 m/min; (**c**) 90 m/min. Feed rate: 0.2 mm/rev, Time: 3 min.

**Figure 11:**
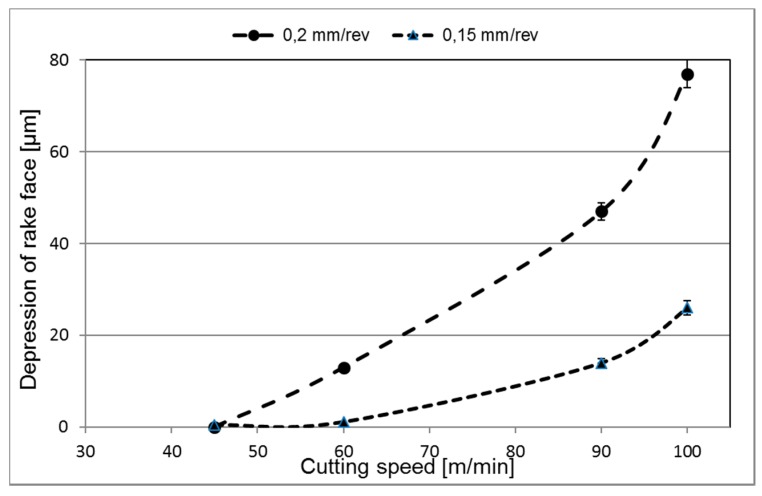
Depression of the rake face in connection to the cutting edge as a function of cutting speed and cutting feed. Time: 3 min.

Figure 9 and Figure 10 show representative cross-sections of worn cutting inserts illustrating the effect of time and cutting speed on the resulting crater and flank wear, respectively. As can be seen, there is a significant increase in crater wear when increasing the cutting speed from 60 m/min to 90 m/min. Also, the SEM micrographs reveal a macroscopic plastic deformation of the cutting edge region resulting in a significant depression of the rake face in connection to the cutting edge. The tendency to depression of the rake face was found to increase with increasing cutting speed, cutting feed and time, see Figure 11. This could be an indication that there is very likely some degree of plastic deformation (depression) of the edge, affecting the present quantifications of the wear rates.

### 3.3. Wear Mechanisms

**Figure 12:**
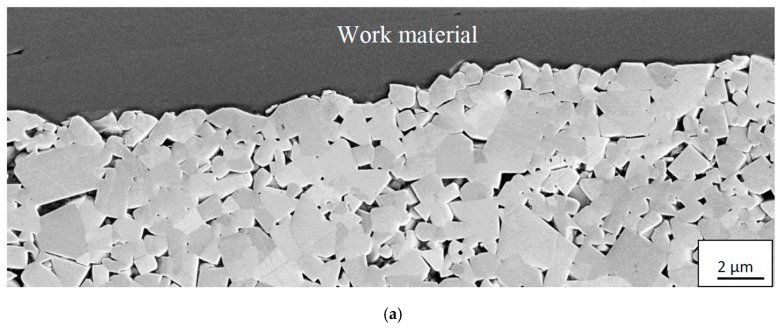

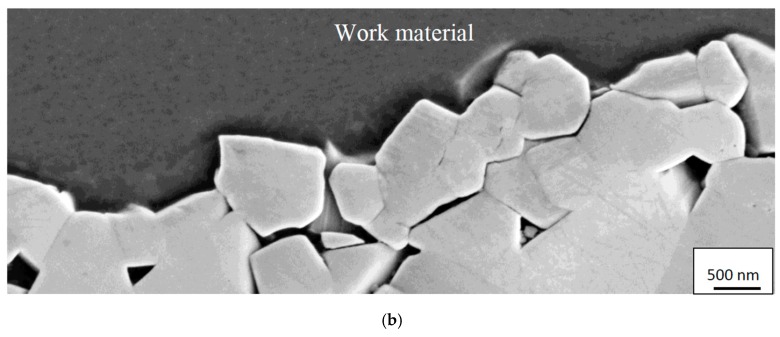
Cross-section of the crater showing the worn rough cemented carbide surface illustrating the presence of an attrition wear mechanism at lower magnification (**a**) and higher magnification (**b**). Attrition wear was found to dominate for combinations of lower cutting feeds/cutting speeds. Chip sliding direction from right to left.

In the following section, the mechanisms controlling the deformation and wear of the cemented carbide under different combinations of cutting feeds/cutting speeds is presented. The analysis is based on a high-resolution SEM, EDS analysis and high-resolution AES analysis of the rake face/chip interface of a number of selected worn cemented carbide inserts. At low cutting feeds/cutting speeds, the worn cemented carbide within the crater displays a relatively rough worn surface underneath the adhered work material indicating that fragments of WC grains or, more probably, individual WC grains had been torn away in contact with the sliding chip, see Figure 12.

**Figure 13:**
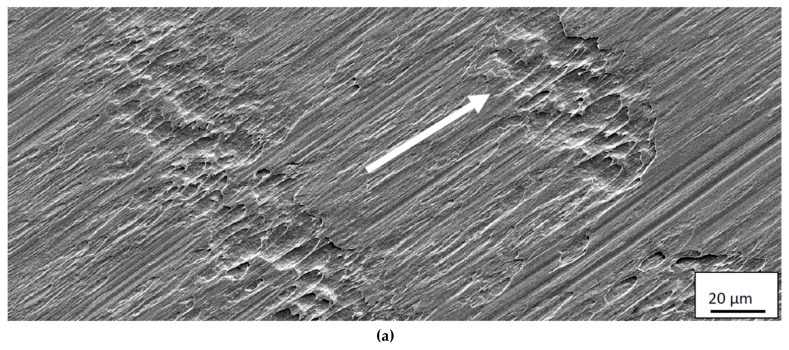

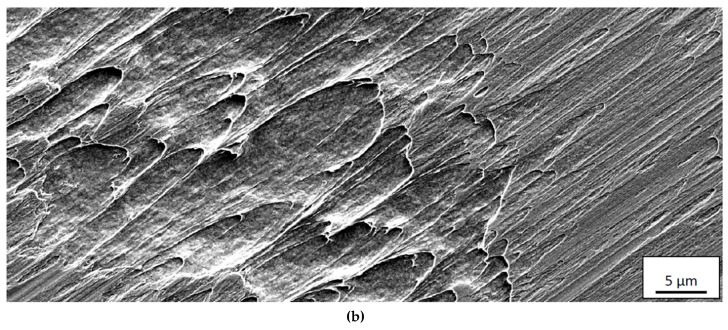
Surface morphology of back side, i.e., the surface in contact with the rake face, of Ti6Al4V chip showing signs of repeated bands revealing the presence of a strong adhesive contact between the chip and the tool rake face at lower magnification (**a**) and higher magnification (**b**). The arrow indicates the chip flow direction.

The relatively small thickness and the pronounced segmented nature of the chip generating cyclic stresses at the interface are believed to promote this type of attrition wear at the rake face/chip interface for low or moderate cutting temperatures. This is supported by the fact that the back side of the chips, i.e., the surface in contact with the rake face, shows a surface morphology with repeated bands showing signs of a strong adhesive contact, see Figure 13. Detailed analyses of the back side of the chips did not reveal any embedded WC particles in the surface. However, the low wear (small wear volumes) of the cemented carbide inserts at low cutting feeds/cutting speeds would make it very difficult (time-consuming) to find these on the back-side of the chips.

**Figure 14:**
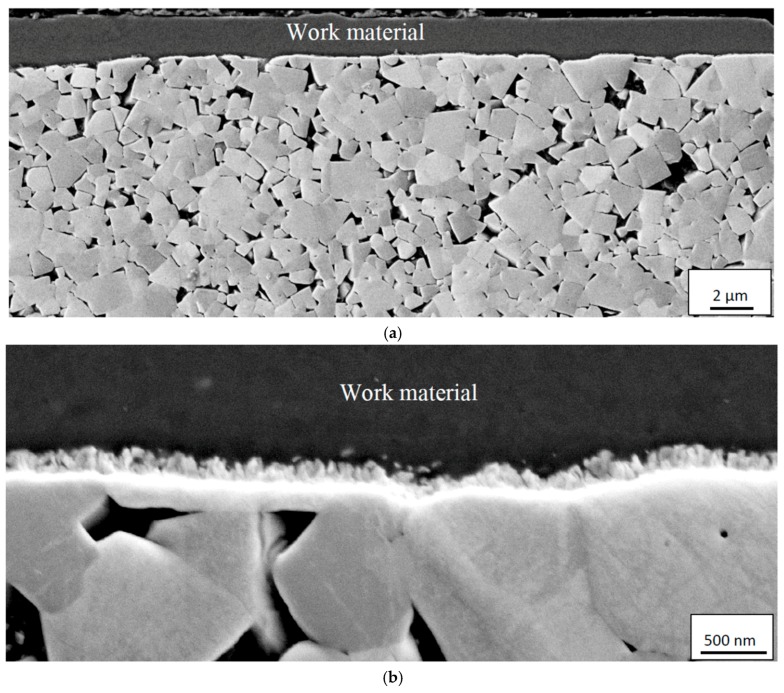
(**a**) Cross-section of the crater showing a smooth cemented carbide surface illustrating the presence of a diffusion wear mechanism. (**b**) Detail showing the presence of the diffusion interfacial layer (white) at the cemented carbide/adhered work material interface. Chip sliding direction from right to left.

**Figure 15:**
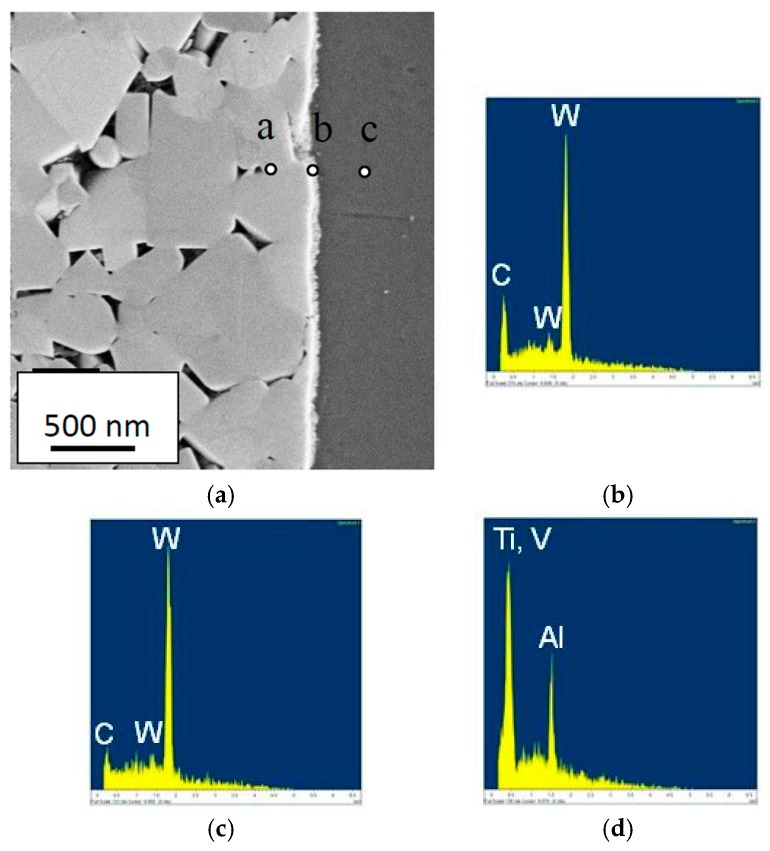
Cross-section of the crater (**a**) showing diffusion layer (white) and high resolution (EDS) spectra (E_0_ = 5 keV) obtained within; a surface WC grain (**b**), the diffusion layer (**c**) and the adhered work material layer (**d**).

**Figure 16:**
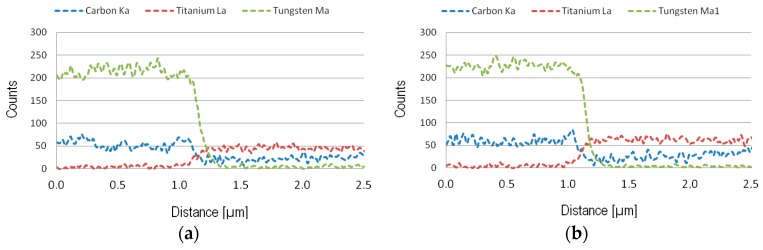
(**a**,**b**) EDS lines scans (E_0_ = 5 keV) illustrating the concentration of C, Ti, and W from the cemented carbide towards the adhered work material. It should be noted that the Co signal shows a line scan profile similar to W and C but at a significantly lower intensity and therefore is excluded.

**Figure 17:**
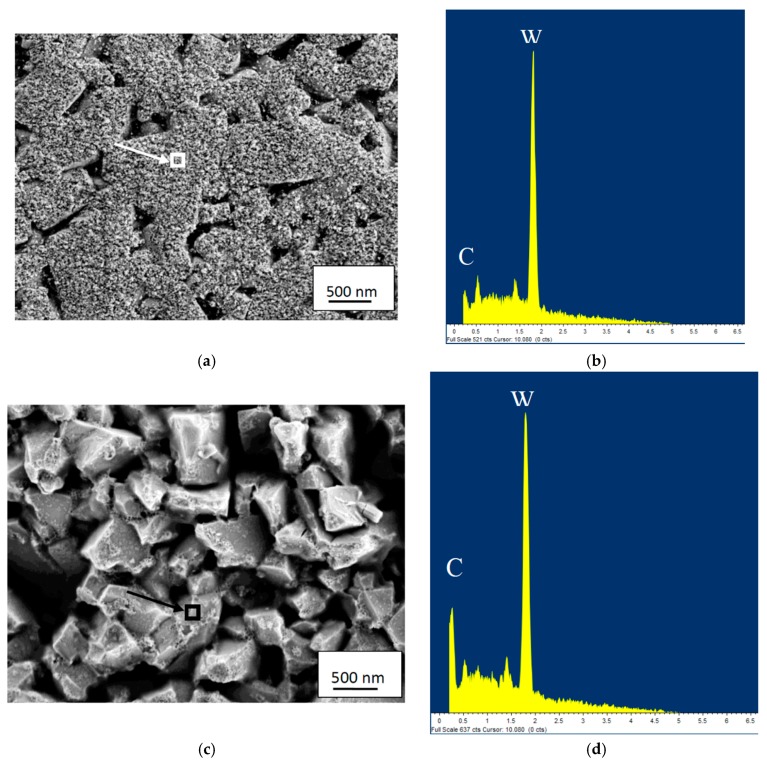
(**a**) Surface morphology of cemented carbide within the crater region showing the presence of a fine grained diffusion layer on the individual WC grains. (**b**) EDS-analysis of the diffusion layer reveals the presence of a WC_1−x_ diffusion zone obtained from the indicated area in Figure 17a. (**c**) Surface morphology of WC grains in the crater region but located within a chipping and not in contact with the chip. (**d**) EDS-analysis of the WC grains reveals a high C signal obtained from the indicated area in Figure 17c.

At higher cutting feeds/cutting speeds, corresponding to higher cutting temperatures at the rake face/chip interface, the worn cemented carbide within the crater displays a very smooth worn surface underneath the adhered work material, see Figure 14. As can be seen, the interface is very sharp without any porosity, demonstrating an intimate adhesion between the work material and the cemented carbide, and reveals the formation of a thin, 100–150 nm, fine grained interfacial layer. The very smooth cemented carbide surface, almost free from pits corresponding to detachment of individual WC grains or clusters of WC grains, indicates the presence of a continuous wear mechanism being active on a very fine, nm, scale. The high chemical reactivity and the low thermal conductivity of Ti6Al4V, resulting in an intimate contact and high temperatures, suggest that wear is controlled by a dissolution-diffusion wear mechanism at the rake face/chip interface, the rate of which is controlled by the temperature. This is supported by EDS spot analysis and EDS line scans across the interface, see Figure 15 and Figure 16, which shows that the white interfacial layer corresponds to a carbon-depleted WC_1−x_ diffusion zone. Also, SEM of the worn cemented carbide within the crater region exposed after etching, see Figure 17, reveals the presence of a fine grained layer on the individual WC grains in the surface. EDS spot analysis of this layer show a significantly lower C content as compared with WC grains in the crater region but located within a chipping and not in contact with the chip (or in slight contact for a short while), and thus supports the presence of a carbon-depleted WC_1−x_ diffusion zone at the interface.

High-resolution SEM and EDS analysis of the flank/work material interface show that also the wear of the flank is strongly dependent on the cutting conditions and that the prevailing wear mechanisms are the same as those at the rake face/chip interface.

**Figure 18:**
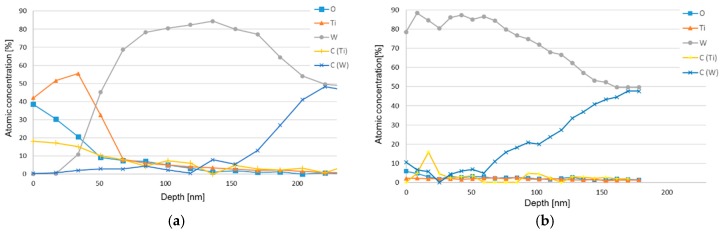
Auger electron spectroscopy (AES) depth profiles obtained from a region within the crater before (**a**) and after (**b**) the removal of the adhered work material layer by etching. The formation of a C-depleted WC zone, 100–150 nm in thickness, is clearly visible in both depth profiles. Also, note the possible formation of TiC in the adhered work material layer in a).

Despite using a relatively low primary electron energy (E_0_ = 5 keV) the interaction volume using EDS is relatively large which makes it difficult to analyze the diffusion zone in detail. Therefore, AES, showing a significantly higher lateral and depth resolution, was used in order to analyze the composition of the diffusion zone in more detail. In the present study, AES depth profiling was used to analyze the chemical composition of the diffusion zone, see Figure 18. In the figure, two different depth profiles are presented. Figure 18a shows a depth profile obtained from an area within the crater showing a thin adhered work material layer while Figure 18b shows a depth profile obtained from an area within the crater were the adhered work material layer had been removed by chemical etching. In both cases, the presence of a C-depleted WC zone, 100–150 nm in thickness, in the interface region is clearly revealed. Also, the depth profile in Figure 18a indicates the formation of TiC within the adhered work material layer just outside the C-depleted WC zone.

The experimental work is described in more detail in another paper, focusing on the systematic investigation of the diffusion degradation [34].

**Figure 19:**
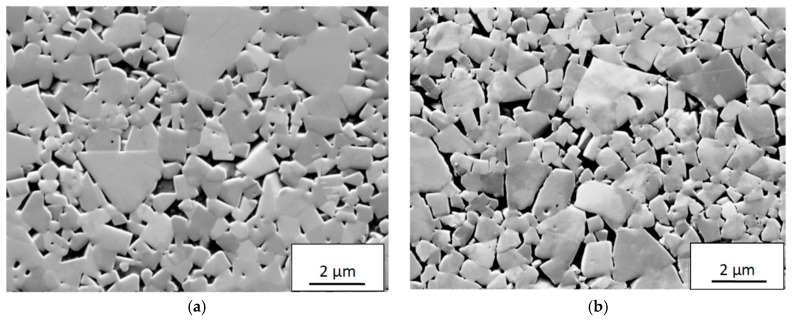
SEM images illustrating the degradation of the cemented carbide structure in the plastically deformed region (20 µm below the surface in the centre of the flank wear land). (**a**) Undeformed WC-Co microstructure; (**b**) deformed WC-Co microstructure. Cutting speed: 90 m/min, feed rate: 0.2 mm/rev., time: 1 min).

SEM and EDS analysis of the deformed microstructure of inserts showing a significant depression of the rake face in connection to the cutting edge revealed that the as-sintered continuous WC skeleton is damaged and has partly broken up within the plastically deformed region, see Figure 19. As can be seen, the Co binder phase has infiltrated the broken grain boundaries forming thin lamella and a more open microstructure. This is in good agreement with Östberg et al. [35,36] who studied the plastic deformation of a WC–10 vol.% Co cemented carbide grade in the turning of a martensitic steel under severe cutting conditions (depth of cut 1 mm, feed 0.3 mm/rev, cutting speeds up to 500 m/min). Micro-Vickers measurements (HV_0.2_) show that the weakening of the WC skeleton results in a lower hardness of the deformed microstructure (1520) as compared with the as-sintered microstructure (1740). It should be noted that the tendency to plastic deformation, i.e., the depression of the rake face in connection to the cutting edge region, observed at combinations of high cutting feed/high cutting speed does not have any significant effect on the wear mechanism, i.e., the weakening of the WC skeleton strength does not result in the detachment of WC grains in the flank and crater wear regions.

**Figure 20:**
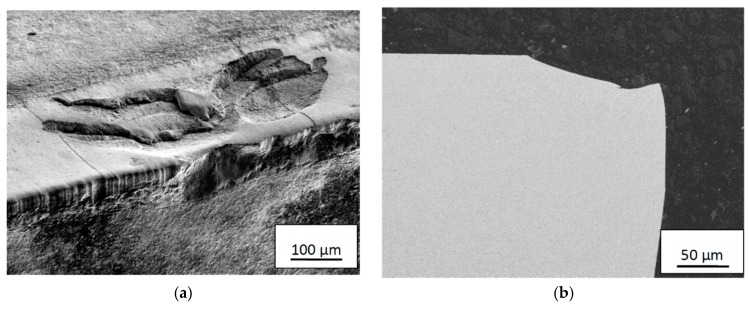
Cracking and macroscopic chipping within the crater (**a**) and fracture of cutting edge region (**b**) obtained at a combination of *v_c_* = 115 m/min and *f_n_* = 0.2 mm/rev after 1–2 min and above 2 min, respectively.

At the most severe combination of cutting feed and cutting speed (in the present study 0.2 mm/rev and 115 m/min) plastic deformation, resulting in a depression of the cutting edge, followed by cracking and macroscopic chipping within the crater was frequently observed after 1–2 min, see Figure 20a. Under these severe cutting conditions cutting times above 2 min frequently results in macroscopic fracture and failure of the cutting edge, see Figure 20b.

**Figure 21:**
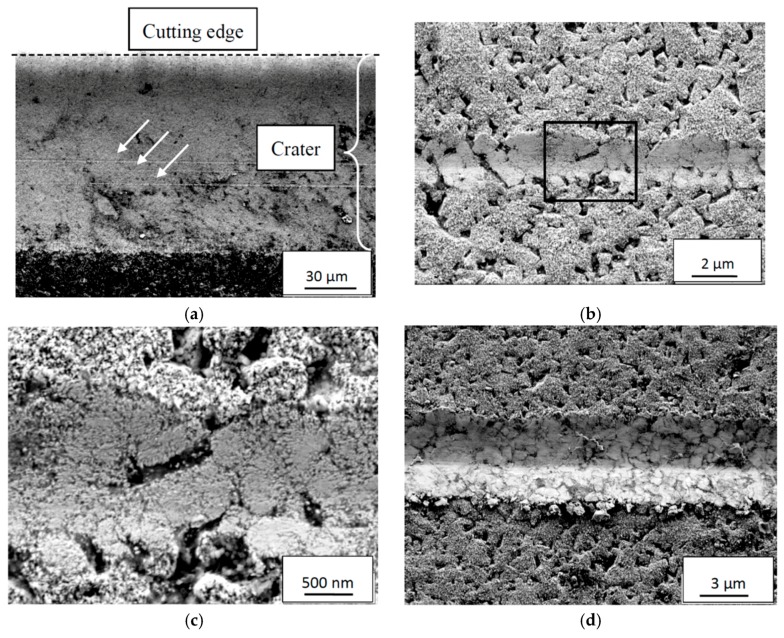
SEM images from the crater area of an etched insert illustrating the scratching characteristics of the WC_1−x_ layer. (**a**) Low magnification overview image showing three scratches within the crater. (**b**) and (**c**) Scratching response at a normal load of 0.01 N; (**d**) Scratching response at a load of 0.05 N.

Scratch testing of the WC_1−x_ layer in the crater area revealed no tendency to adhesive failure. At low normal loads using a sharp diamond stylus (radius 7.5 µm), see Figure 21, post-test SEM studies of the scratches revealed no tendency to adhesive failure of the WC_1−x_ layer. At low normal load, 0.01 N, the layer displayed a low cohesive strength where the individual columnar grains WC_1-x_ layer tend to detach and smear out in the scratch track, see Figure 21c. A higher normal load, 0.05 N, see Figure 21d, results in a more macroscopic deformation of the cemented carbide but no tendency to extensive failure of the cemented carbide composite.

## 4. Discussion

The results of the present study indicate that both crater and flank wear are controlled by mainly two different wear mechanisms. At combinations of lower cutting feeds or cutting speeds attrition wear will dominate while at combinations of higher cutting feeds or cutting speeds diffusion wear will dominate. To agree with the models by Archard [3] and Usui et al. [4], the former wear mechanism should result in a linear increasing wear while the latter should result in a linear with higher slope or exponential increasing wear with increasing cutting feed or cutting speed. Our estimated wear rates are in agreement with these predicted behaviours. The wear measurements show linear progressions with time/distance for all feed rates and velocities, as illustrated in Figure 7, regardless of the number of flanges (1–4) that were used for each measurement. These linear progressions give some indication that the wear rates reach steady-states quickly during cuts. If the wear is connected to temperature, these results could also indicate that the cutting zone temperature is relatively constant during measurements. It should be noted that evidence of a diffusion layer can be obtained by high-resolution SEM of polished cross-sections or by high-resolution SEM of the worn craters in inserts being etched in order to remove the adhered work material. Some indications of plastic depression of the cutting edge have been observed. A simultaneous plastic deformation mechanism would lead to an overestimation of the worn volume, which leaves the possibility that our estimated wear rates are higher than what diffusion or attrition wear alone would result in. We have included information about the edge depression for different cutting conditions, allowing for a possible refinement of the data, which could e.g., combine the present measurements with a quantification of the plastic deformation in the binder discussed by Kaplan et al. [6]. For the only possible point of comparison at present, a recalculation of the wear rate for *v_c_* 70 m/min and *f_n_* 0.2 mm/rev in Figure 8, assuming a contact area of 3 mm width (flange width) and 0.4 mm length (double the feed), gives a wear rate in the same order of magnitude as the one found by Odelros et al. [7] although about 50% higher. To further link the wear rate measurements from the present work with a rate-limiting mechanism, estimations of cutting zone temperatures for the different machining operations have to be made. This has been considered outside the scope of the present work.

High-resolution SEM, EDS line scan analysis as well as high-resolution AES depth profiling of the diffusion layer reveal the formation of an approximately 100–150 nm thick carbon-depleted WC_1−x_ layer at the cemented carbide/Ti6Al4V interface. This is in good agreement with recent findings obtained by Kaplan et al, Odelros et al and Latteman et al., see referrences [6,7,37]. Also, recent work based on static diffusion couples were the chemical reaction between cemented carbide and Ti-alloys at elevated temperatures agree well with the present findings [8,38].

**Figure 22:**
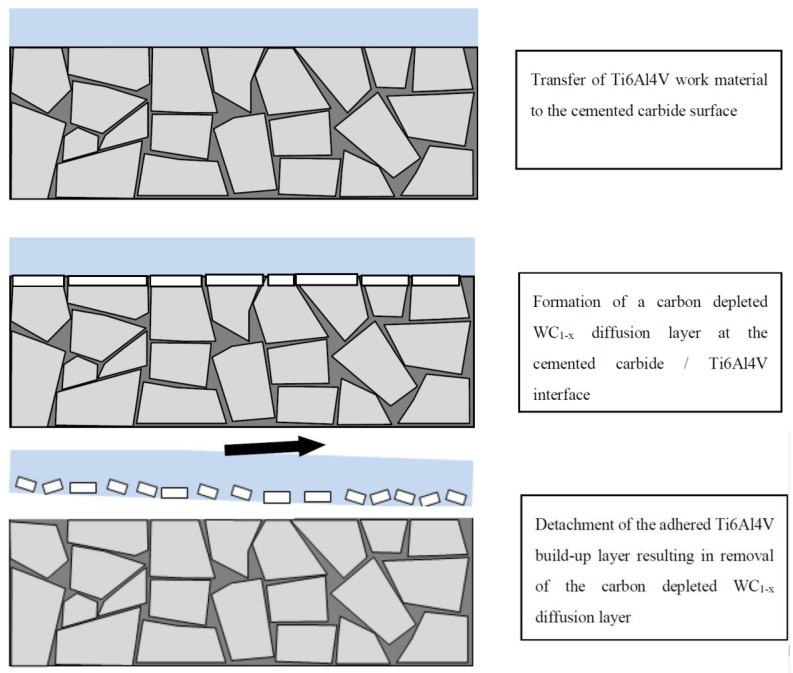
Schematic illustrating the degradation and wear of cemented carbide during turning of Ti6Al4V. The lower strength of the WC_1−x_ diffusion layer at the cemented carbide/Ti6Al4V interface will promote detachment of the adhered build-up layer, including part of the WC_1−x_ diffusion layer.

**Figure 23:**
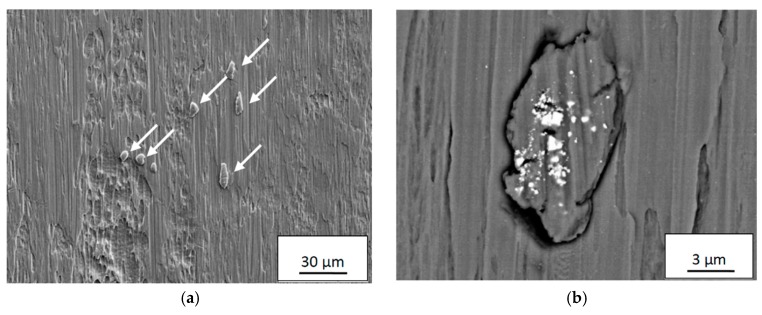

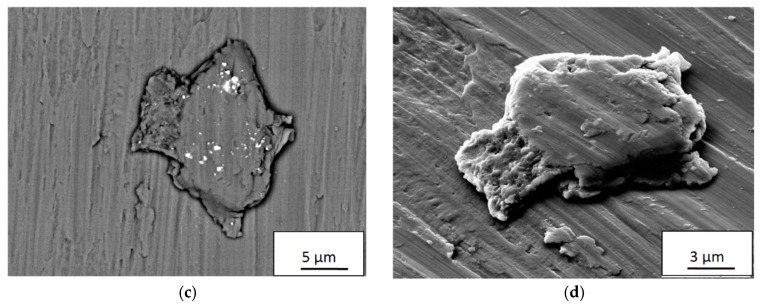
(**a**) Fragments (indicated by arrows) of Ti6Al4V build-up layers containing fine debris of the diffusion WC_1−x_ layer found on the back-side of Ti6Al4V chips. (**b**) and (**c**)High magnification SEM BEI micrographs showing the presence of WC_1−x_ debris in the surface of the detached build-up layer fragments. (**d**) SEM tilted-view micrograph of the build-up layer fragment in Figure **c**.

Based on the present findings, it is believed that the diffusion of carbon from the WC-phase, resulting in a carbon-depleted WC_1−x_ layer with reduced mechanical strength, will promote detachment of the adhered build-up layers of work material on the rake and flank surfaces when exposed to high shear stresses. If we assume that the WC_1−x_ layer will stick to the detached build-up layers, i.e., that cracking will take place along the WC/WC_1−x_ interface or within the WC_1−x_ layer as soon as it reaches a critical thickness, the detachment of the build-up layer will expose the WC phase for the work material which immediately will adhere to the cemented carbide surface promoting the diffusion-controlled wear of the cemented carbide to continue. Consequently, the diffusion rate of carbon into the build-up layer and the removal rate of the WC_1−x_ layer determine the wear rate of the cemented carbide tool material, see Figure 22. The above wear model is supported by the results from detailed SEM and EDS analysis of the back-side of the chips which revealed the presence of Ti6Al4V build up layer fragments, 10–20 µm in diameter, see Figure 23, in the chip surface. AES depth profile is performed on the fragments and it has been proved that the fragments are WC_1−x_. The result is included in another paper [34]. At higher magnification, see Figure 23b–d, a high amount of fine debris originating from the diffusion layer can be seen embedded in the surface of the fragments. In contrast, the surrounding chip surface do not contain any wear fragments originating from the cemented carbide insert.

## 5. Conclusions

The wear and wear rate mechanisms of cemented carbide in the turning of Ti6Al4V have been studied under well-controlled conditions. Based on the obtained results the following conclusions can be drawn;

- Crater and flank wear of the cemented carbide inserts increase with increasing temperature at the cutting zone, i.e., with increasing cutting speed and feed.
- Crater and flank wear for combinations of low cutting speeds and feeds, are controlled by an attrition wear mechanism while diffusion wear mechanism was found to control the wear in the case of combination of media to high cutting speeds and feeds.
- At low cutting speeds or feeds, the worn cemented carbide displays a rough worn surface underneath the adhered work material indicating that fragments of WC grains or individual WC grains had been torn away when the adhered work material layer is removed by the chip.
- At medium to high cutting speeds or feeds, the worn cemented carbide displays a very smooth worn surface underneath the adhered work material. High-resolution SEM, EDS and AES depth profile analysis reveal the formation of an approximately 100–150 nm thick carbon-depleted WC-layer at the cemented carbide/Ti6Al4V interface due to the diffusion of carbon into the adhered work material on the rake and flank surfaces.
- Detailed SEM and EDS analysis of the back-side of the chips reveals the presence of Ti6Al4V build up layer fragments containing fine debris originating from the carbon-depleted WC-layer.
- Severe plastic deformation and macro cracking/fracture of the cutting edge region which may result in catastrophic failure is due to combinations of high cutting speed and feed.

## Figures and Tables

**Table 1:** Chemical composition (in weight %) and hardness of the work-piece material Ti6Al4V according to the material specification provided by material supplier Timet.

WC [vol%]	Co [vol%]	WC Grain Size [µm]	Hardness, HV_3_
89.8	10.2	1	1580

**Table 2:** Chemical composition (in weight %) and hardness of the work-piece material Ti6Al4V according to the material specification provided by material supplier Timet.

Al	V	Fe	C	O	N	Y	Ti	Hardness, HV_5_
6.425	3.970	0.155	0.019	0.190	0.006	<0.001	Bal.	300
